# Notes on Preparations for the Sick

**Published:** 1899-12

**Authors:** 


### Notes on Preparations for the Sick.

**Bromidia.**—Battle & Co., Saint Louis.—This hypnotic, manufactured in Paris, is an excellent combination ready made for those who cannot write a prescription or dispense one. It is composed of well-known drugs in convenient and palatable combination, and it usually acts very efficiently in cases where a chloral and bromide sedative is indicated.

**Ferru-Cocoa.**—Ferru-Cocoa Manufacturing Co., London.—A cocoa containing iron, kola nut, and malt is a combination likely to be useful, not only to the anæmic girl, but to all varieties of persons who require to be fit for any laborious occupation.

**Soloids: Sodium Chloride; Sodium Chloride and Sodium Sulphate; Sodium Chloride Compound; Lead Subacetate; Lead and Opium.**—BURROUGHS, WELLCOME & Co.—The sodium chloride preparations are intended for solution in sterilised water, and to be then used as intravenous injections. The advantages of having lead soloids in a portable form are obvious.

---

**The "Alîoné" Clothing.**—ALîONÉ COMPANY, London.—These articles of clothing have been devised for the purpose of facilitating the dressing of infants and invalids without turning or disturbing them. The invalid night-dress can be opened at back, front, and sleeves.

---

**The "Bella-Wattee Patent Teapot."**—THE BELLA-WATTEE COMPANY, London.—This is so devised that, after a few minutes infusion, the leaves can be raised into the lid of the pot and there fixed quite clear of the liquid. The arrangement appears to be quite a success, and is well spoken of by those who have tried it.

---

